# Recent Tobacco Smoking is Associated with Poor HIV Medical Outcomes Among HIV-Infected Individuals in New York

**DOI:** 10.1007/s10461-015-1273-x

**Published:** 2016-02-02

**Authors:** Stephen J. Hile, Matthew B. Feldman, Emily R. Alexy, Mary K. Irvine

**Affiliations:** New York City Department of Health and Mental Hygiene, Bureau of HIV/AIDS Prevention and Control, 42-09 28th Street, Queens, NY 11101-4132 USA

**Keywords:** Tobacco smoking, HIV, Viral load, CD4 cell count

## Abstract

Tobacco smoking is associated with adverse health effects among people living with HIV (PLWH), including a higher risk of cancer and cardiovascular problems. Further, there is evidence that PLWH are two to three times more likely to smoke than the general population. The aim of this study was to examine the association between tobacco smoking and biomarkers of HIV disease progression, including unsuppressed viral load (viral load >200 copies/mL) and low CD4 cell count (<200 cells/mm^3^). Recent tobacco smoking was reported by 40 % (n = 5942) of 14,713 PLWH enrolled in Ryan White Part A programs in the New York City metropolitan area. In multivariate analyses controlling for sociodemographic and clinical characteristics, recent tobacco smoking was independently associated with unsuppressed viral load (AOR = 1.38, CI 1.26–1.50) and low CD4 cell count (AOR = 1.12, CI 1.01–1.24). Findings suggest the importance of routine assessments of tobacco use in clinical care settings for PLWH.

## Introduction

Cigarette smoking is the leading cause of preventable mortality in the United States [1]. Between 2005 and 2009, roughly 480,000 deaths occurred each year due to cigarette smoking and/or exposure to tobacco smoke. Although the prevalence of smoking in the general population has decreased from 42 % in 1965 to 18 % in 2012 [1], a high prevalence of smoking persists among people living with HIV (PLWH). Studies have reported smoking prevalence estimates for PLWH that are approximately two to three times greater than those for the general population, ranging from 40.5 to 74 % [2–7].

In the era of widely available and effective antiretroviral therapy (ART), the life expectancy of HIV-infected individuals can approach that of individuals who are uninfected [8, 9]. Recent studies, however, have found that smoking may offset these increases in survival among PLWH. In a nationwide population-based cohort study of PLWH in Denmark, both AIDS-related and non-AIDS-related mortality rates were higher among smokers compared to non-smokers [8]. Other studies with PLWH have demonstrated associations between smoking and an increased risk of health conditions including cancer [10], bacterial pneumonia, opportunistic infections [11], and cardiovascular disease [12, 13]. As the population of PLWH ages, it is increasingly important to understand how behaviors, such as smoking, impact HIV disease progression.

Smoking has also been linked with decreases in CD4 cell count [14, 15]. Feldman et al. [16] observed that, after an initial increase in CD4 cell count and percentage among HIV-infected smokers, both values eventually decreased below the levels among HIV-infected non-smokers. However, several studies have found no significant relationship between smoking and CD4 cell count [3, 17–21].

The few studies that have examined the effect of smoking on viral suppression have yielded similarly mixed results. In a cross-sectional study of 56 HIV-infected and uninfected women, Wojna et al. [15] observed an association between current smoking and higher viral load. Feldman et al. [16] also found that smokers had a lower chance of achieving a viral response (i.e., a decrease in HIV-1 RNA to ≤80 copies/mL), and had a greater chance of developing viral failure (i.e., an HIV-1 RNA increase to >1000 copies/mL after an initial viral response), in a longitudinal cohort study of low-income women receiving ART. Miguez-Burbano [22] found that viral responses to ART were attenuated in cigarette smokers. However, one study found no significant relationship between smoking and viral load [23].

In addition to the mixed results on the relationship between smoking and biomarkers of HIV disease progression, existing research also has methodological concerns. First, several prior studies did not adequately control for potentially important confounders, including ART status [15, 17–21] and substance use [15, 17–19, 22]. There is evidence that both of these variables are associated with smoking and/or HIV medical outcomes [16, 22, 24–28]. Second, many of the prior studies also used small samples (i.e., n < 250), which limit the power of those analyses [15, 17, 21, 22].

This study improves on prior research by controlling for ART prescription status and substance use, in addition to using a large sample of PLWH (n = 14,713). To our knowledge, this is the largest sample to date for a study of tobacco smoking and biomarkers of HIV disease progression in a major metropolitan area of the United States. The aim of this analysis was to examine the association between recent tobacco smoking and biomarkers of HIV disease progression (unsuppressed viral load, low CD4 cell count) among PLWH enrolled in Ryan White Part A services in the New York Eligible Metropolitan Area (NY EMA).

## Methods

### Client Population

Of 24,114 HIV-infected Ryan White clients who were enrolled in Ryan White Part A services in the NY EMA (including New York City and the counties of Putnam, Rockland, and Westchester) between 11/1/2010 and 9/20/2013, there were 19,042 individuals over the age of 18 who had completed at least one valid substance use assessment during that period of time. Of those individuals, 17,554 matched to the New York City HIV Surveillance Registry (“the Registry”) and had at least one documented viral load and/or CD4 cell count in the Registry. The final sample for this analysis included 14,713 HIV-infected individuals who had evidence of a viral load and/or CD4 cell count in the Registry with a test date within the 3-month period prior to the most recent substance use assessment. Substance use assessments were excluded from the analysis if the response to the question about recent tobacco use was missing or “declined” or if the client indicated tobacco use through means other than smoking (e.g., chewing tobacco). If a client completed more than one qualifying substance use assessment, the most recent assessment during the study period was used.

Client characteristics were compared between the final eligible sample and clients who matched to the Registry, but did not have evidence of a viral load or CD4 cell count with a test date within the 3-month period prior to the most recent substance use assessment (n = 2841). Clients in the analysis sample were significantly more likely to be Hispanic, living below 100 % of the federal poverty level, unstably or temporarily housed, prescribed ART, and educated below a high school graduate level, as compared with those excluded for not having a lab test within the specified window. Eligible clients were also more likely to have had at least one alcoholic drink during the last 3 months and a more recent date of HIV diagnosis.

### Data

New York State mandates the reporting of all names and diagnoses of HIV and AIDS, all HIV-related illness, all positive Western blot tests for HIV antibody, and all viral load and CD4 cell count values [29]. This information is collected through the Registry, an Electronic HIV/AIDS Reporting System (eHARS)-based surveillance database that contains comprehensive information on HIV diagnoses and HIV-related laboratory results from medical providers in New York City. It is continuously updated with new de-duplicated data. The Registry was used as the data source for viral loads, CD4 cell counts, and dates of HIV diagnosis. Demographic and behavioral data were collected through the Electronic System for HIV/AIDS Reporting and Evaluation (eSHARE). eSHARE is an internally developed, secure, web-based local health department system for contractually required reporting of client-level demographic, psychosocial, clinical, and service-related data by Ryan White Part A-funded HIV service providers in the NY EMA. Ryan White Part A in the NY EMA funds clinical and supportive services for PLWH with a household income below 435 % of the federal poverty level.

The collection of fully identified data (including client names) from HIV care and treatment programs in eSHARE permits de-duplication and merging of programmatic data with the Registry. eSHARE data were merged with viral load, CD4, and HIV diagnosis date data that were considered to be complete in the Registry as of 9/30/2013. For the purpose of this analysis, the datasets were de-identified following the merge. The New York City Department of Health and Mental Hygiene (NYC DOHMH) Institutional Review Board deemed this analysis to be exempt from the board’s review, since use of the de-identified, limited datasets would pose minimal risk to clients’ privacy and data confidentiality.

### Measures

#### Tobacco Smoking

A question on the eSHARE substance use assessment about tobacco use in the past 3 months was analyzed to assess recent tobacco use (yes/no). Clients were classified as having recently smoked tobacco if they responded “yes” to this question and indicated “smoking” as the route of administration.

#### HIV Medical Outcomes

For each participant, the viral load and/or CD4 cell count closest to the date of the most recent substance use assessment (and within the 3-month period prior to that assessment) were selected from the Registry. A low CD4 count was defined as <200 cells/mm^3^, which represents the CD4 cell count at which most opportunistic infections are likely to occur [30–32], and at which Stage 3 HIV (AIDS) is diagnosed for persons age six and over [33]. Unsuppressed viral load was defined as an HIV-1 RNA >200 copies/mL, which is the threshold for virologic failure [30].

#### Sociodemographic and Clinical Characteristics

Covariates for this analysis were consistent with those of prior studies of tobacco smoking among PLWH [15–18, 20–23], and included current self-identified gender (male vs. female or transgender), race/ethnicity (white vs. black, Hispanic or other), age (<30 vs. 30–49 or 50+), education (< vs. ≥high school diploma/GED), primary language (other vs. English), country of birth (USA/US territory vs. other country), income (≥ vs. <100 % of the federal poverty level), housing situation (stable vs. temporary or unstable housing), ART prescription status (prescribed vs. not prescribed ART), recent hard drug use (any vs. no cocaine/crack, heroin, methamphetamine, hallucinogen, or recreational prescription drug use in the past 3 months), and number of years since HIV diagnosis.

Prior studies have found that tobacco smoking is associated with heavy alcohol consumption among PLWH [3, 34–36]. The substance use assessment allowed us to determine if a participant had consumed at least one alcoholic beverage in the last 3 months, but inconsistent data collection on this substance prevented us from reliably measuring the frequency or quantity of alcohol consumption. We were therefore unable to distinguish heavy from other alcohol use. Due to this limitation, recent or lifetime alcohol use was not included as a covariate in our analysis.

### Data Analysis

Chi square tests were used to examine the differences between HIV-infected individuals with and without recent smoking. Bivariate analyses of the relationship between each covariate and biomarkers of HIV disease progression were conducted using logistic regression to estimate odds ratios. Multivariate logistic regression models were then used to identify the variables that were independently associated with low CD4 cell count and unsuppressed viral load. Variables that were statistically significant (*P* < .05) in bivariate analyses were included in the multivariate models. In the multivariate models for unsuppressed viral load and low CD4 count, interaction tests were performed to determine if recent drug use modified the effect of recent tobacco smoking.

Due to the unavailability of reliable data on frequency and quantity of recent alcohol use, we conducted sensitivity analyses by rerunning the multivariate regression models for CD4 cell count and viral load suppression while restricting the sample to clients who reported no alcohol use in the last 3 months. Results are shown as adjusted odds ratios (AOR) with their corresponding 95 % confidence intervals (CIs). Data were analyzed using SAS statistical software version 9.2 (SAS Institute Inc., Cary, NC, USA).

## Results

In a sample of 14,713 PLWH who received Ryan White Part A-funded services, 40 % (n = 5942) reported recent tobacco smoking. Recent smokers were significantly more likely than non-smokers to be black, over 30 years old, living below 100 % of the federal poverty level, English-speaking, born in the USA or a US territory, unstably or temporarily housed, educated below a high school graduate level, and virally unsuppressed. Recent smokers were also more likely to have a low CD4 cell count, to have consumed at least one alcoholic drink in the last 3 months, to have an earlier date of HIV diagnosis, and to not be prescribed ART (Table 1).

**Table 1:** Sociodemographic and clinical characteristic differences between PLWH receiving Ryan White Part A services in the New York Eligible Metropolitan Area with and without recent tobacco smoking (N = 14,713)

Characteristic^a^	Recent tobacco smoking N = 5942 (40 %)	No recent tobacco smoking N = 8771 (60 %)	*P* value
	n (col %)	n (col %)	
Female (vs. male)	1903 (32.1 %)	2955 (33.7 %)	.004
Transgender (vs. male)	117 (2.0 %)	121 (1.4 %)	
Black/African American (vs. white)	3322 (56.1 %)	4473 (51.2 %)	<.001
Hispanic (vs. white)	1930 (32.6 %)	3169 (36.3 %)	
Other (vs. white)	163 (2.8 %)	357 (4.1 %)	
30–49 (vs. <30)	2764 (46.5 %)	3876 (44.2 %)	<.001
50 + (vs. <30)	2566 (43.2 %)	3781 (43.1 %)	
≥High school diploma	3191 (54.7 %)	5074 (60.9 %)	<.001
English speaking	5177 (87.2 %)	6456 (73.7 %)	<.001
Born outside USA/US territory	487 (8.2 %)	2567 (29.6 %)	<.001
Income ≥100 % federal poverty level	786 (14.5 %)	1730 (22.7 %)	<.001
Temporary housing (vs. stable)	927 (15.9 %)	978 (11.5 %)	<.001
Unstable housing (vs. stable)	1407 (24.1 %)	1025 (12.0 %)	
Not prescribed ART	1040 (17.5 %)	1264 (14.8 %)	<.001
Recent drug use	1632 (28.5 %)	480 (5.6 %)	<.001
Recent alcohol use^b^	2788 (47.8 %)	1852 (21.3 %)	<.001
Low CD4 cell counts^c^	1321 (23.4 %)	1506 (18.1 %)	<.001
Unsuppressed viral load^d^	2658 (46.4 %)	2688 (31.8 %)	<.001
Years since HIV diagnosis [M (SD)]	12.6 (7.2)	11.4 (7.2)	<.001

^a^Missing responses within covariates were not included in analyses

^b^Recent alcohol use is defined as having one or more drinks in the 3 months prior to the substance use assessment

^c^<200 cells/mm^3^

^d^>200 copies/mL

In multivariate analyses controlling for sociodemographic and clinical characteristics, there was a significant independent association between recent tobacco smoking and both unsuppressed viral load (Table 2; AOR = 1.38, CI 1.26–1.50) and low CD4 cell count (Table 3; AOR = 1.12, CI 1.01–1.24). Tests of interaction between recent tobacco smoking and recent hard drug use were not significant in either multivariate model. In the sensitivity analyses in which we restricted the sample to only those who did not report recent alcohol use in the last 3 months (n = 10,681), we found that there was still an independent relationship between recent tobacco smoking and unsuppressed viral load (AOR = 1.35, CI 1.21–1.50); however, the relationship between recent tobacco smoking and low CD4 cell count was no longer significant at the multivariate level (AOR = 1.11, CI 0.98–1.25).

**Table 2:** Factors associated with unsuppressed HIV-1 RNA among PLWH receiving Ryan White Part A services in the New York Eligible Metropolitan Area (N = 14,178)

Characteristic^a^	Viral load >200^b^ N = 5346 (40 %)	Viral Load ≤200^b^ N = 8832 (40 %)	OR (0.95 CI)	AOR (0.95 CI)
	n (col %)	n (col %)		
Recent tobacco smoking	2658 (49.7 %)	3070 (34.8 %)	1.86 (1.73–1.99)	1.38 (1.26–1.50)
Female (vs. male)	1731 (32.4 %)	2926 (33.2 %)	0.97 (0.91–1.05)	1.00 (0.91, 1.09)
Transgender (vs. male)	107 (2.0 %)	128 (1.5 %)	1.38 (1.06–1.79)	1.03 (0.76, 1.41)
Black/African American (vs. white)	3080 (57.8 %)	4432 (50.4 %)	1.54 (1.35–1.76)	1.37 (1.17–1.61)
Hispanic (vs. white)	1720 (32.3 %)	3198 (36.4 %)	1.19 (1.04–1.37)	1.20 (1.01–1.42)
Other (vs. white)	157 (3.0 %)	353 (4.0 %)	0.99 (0.79–1.24)	0.99 (0.75–1.30)
30–49 (vs. <30)	2674 (50.0 %)	3739 (42.3 %)	0.67 (0.60–0.75)	0.74 (0.65–0.84)
50+ (vs. <30)	1811 (33.9 %)	4286 (48.5 %)	0.40 (0.36–0.44)	0.46 (0.40–0.53)
≥High school diploma	2928 (56.4 %)	5053 (59.6 %)	0.88 (0.82–0.94)	0.93 (0.85–1.01)
English speaking	4466 (83.6 %)	6739 (76.3 %)	1.58 (1.45–1.72)	1.07 (0.94–1.23)
Born outside USA/US territory	769 (14.5 %)	2189 (25.0 %)	0.51 (0.46–0.56)	0.67 (0.59–0.76)
Income ≥100 % federal poverty level	680 (14.4 %)	1754 (22.3 %)	0.59 (0.53–0.65)	0.75 (0.67–0.84)
Temporary housing (vs. stable)	758 (14.5 %)	1065 (12.3 %)	1.46 (1.32–1.61)	1.05 (0.93–1.19)
Unstable housing (vs. stable)	1272 (24.4 %)	1072 (12.4 %)	2.43 (2.22–2.66)	1.72 (1.54–1.92)
Not prescribed ART	1457 (27.5 %)	741 (8.6 %)	4.05 (3.68–4.46)	3.52 (3.14–3.94)
Recent drug use	1158 (22.3 %)	853 (9.9 %)	2.62 (2.38–2.88)	1.94 (1.73–2.17)
Years since HIV diagnosis [M (SD)]	11.3 (7.4)	12.2 (7.1)	0.98 (0.98–0.99)	1.00 (0.99–1.01)

^a^Missing responses within covariates were not included in analyses

^b^Copies/mL

**Table 3:** Factors associated with a low CD4 cell count among PLWH receiving Ryan White Part A services in the New York Eligible Metropolitan Area (N = 13,955)

Characteristic^a^	CD4 < 200^b^ N = 2827	CD4 ≥ 200^b^ N = 11,128	OR (0.95 CI)	AOR (0.95 CI)
	n (col %)	n (col %)		
Recent tobacco smoking	1321 (46.7 %)	4321 (38.8 %)	1.38 (1.27–1.50)	1.12 (1.01–1.24)
Female (vs. male)	906 (31.1 %)	3710 (33.4 %)	0.94 (0.86–1.03)	–
Transgender (vs. male)	40 (1.4 %)	183 (1.7 %)	0.84 (0.60–1.89)	–
Black/African American (vs. white)	1630 (57.9 %)	5804 (52.4 %)	1.47 (1.25–1.74)	1.28 (1.07–1.54)
Hispanic (vs. white)	927 (32.9 %)	3875 (35.0 %)	1.26 (1.06–1.49)	1.15 (0.94–1.40)
Other (vs. white)	70 (2.5 %)	420 (3.8 %)	0.88 (0.65–1.18)	0.89 (0.63–1.25)
30–49 (vs. <30)	1345 (47.6 %)	4917 (44.2 %)	1.33 (1.15–1.53)	1.15 (0.98–1.36)
50 + (vs. <30)	1200 (42.5 %)	4845 (43.5 %)	1.20 (1.04–1.38)	0.93 (0.78–1.10)
≥High school diploma	1483 (54.1 %)	6361 (59.4 %)	0.80 (0.74–0.88)	0.85 (0.77–0.94)
English speaking	2337 (82.7 %)	8732 (78.5 %)	1.31 (1.17–1.45)	1.02 (0.87–1.19)
Born outside USA/US territory	413 (14.7 %)	2460 (22.3 %)	0.6 (0.54–0.67)	0.8 (0.69–0.94)
Income ≥100 % federal poverty level	407 (16.3 %)	1973 (20.0 %)	0.78 (0.69–0.88)	0.9 (0.79–1.02)
Temporary housing (vs. stable)	376 (13.6 %)	1430 (13.1 %)	1.13 (1.00–1.28)	1.06 (0.92–1.22)
Unstable housing (vs. stable)	587 (21.2 %)	1733 (15.9 %)	1.45 (1.31–1.62)	1.21 (1.07–1.37)
Not prescribed ART	490 (17.5 %)	1677 (15.4 %)	1.17 (1.04–1.30)	1.17 (1.03–1.33)
Recent drug use	533 (19.4 %)	1451 (13.4 %)	1.17 (1.04–1.30)	1.32 (1.17–1.50)
Years since HIV diagnosis [M (SD)]	13.2 (7.1)	11.6 (7.2)	1.03 (1.03–1.04)	1.03 (1.03–1.04)

^a^Missing responses within covariates were not included in analyses

^b^cells/mm^3^

## Discussion

We found a significant relationship between recent tobacco smoking and low CD4 cell count and unsuppressed viral load, which is consistent with prior studies that also detected a significant association between smoking and biomarkers of HIV disease progression [14–16, 22]. To date, the findings for this relationship have been mixed; therefore, this analysis represents an important contribution to the existing knowledge base on smoking among PLWH.

A significantly higher proportion of individuals who reported recent smoking, as compared to non-smokers, also reported recent hard drug use (28.5 vs. 5.6 %). Further, the association between recent hard drug use and both unsuppressed viral load and low CD4 cell count remained significant in the multivariate models. Our analysis found no evidence of interaction between recent tobacco smoking and recent hard drug use in either multivariate model. Prior studies have similarly found that a high proportion of PLWH who smoke also use illicit drugs [34, 35], and that current illicit drug use is independently associated with smoking in this population [34, 37, 38]. There is also prior evidence of a significant association between illicit drug use and HIV medical outcomes [24, 39, 40], even when controlling for ART status [25–27, 41]. Together, these findings may support the existence of a direct physiological pathway by which drug use is associated with HIV medical outcomes, independent of the effect of ART adherence [42, 43]. Future analyses should examine the potential role of drug use in the relationships between tobacco smoking, ART adherence, and biomarkers of HIV disease progression.

Recent tobacco smoking remained independently associated with low CD4 cell count and unsuppressed viral load even when controlling for ART prescription status, a finding that is consistent with prior studies [16, 22]. These findings may support the hypothesis that tobacco smoking has a direct physiological effect on HIV-related health [44]. For example, there is evidence that nicotine may suppress immune functioning in several ways, including impairing T-cell proliferation by disrupting the cell’s ability to attach to antigen cells [44, 45]. While there may be biological mechanisms by which smoking affects the immune system, smoking may also be linked to biomarkers of HIV disease progression through lower ART adherence, which could not be measured for this analysis. There is evidence that HIV-infected smokers are less adherent to ART medications than HIV-infected nonsmokers [21, 28]. Low adherence is also associated with alcohol use and substance use [46–48]. However, to our knowledge, no studies to date have specifically examined whether ART adherence accounts for the relationship between tobacco smoking and low CD4 cell count and unsuppressed viral load.

The findings of this analysis underscore the need to address smoking in the context of clinical and supportive services for PLWH. There are unique barriers to smoking cessation for PLWH, including high rates of substance use, lack of strong social support networks, and the use of tobacco to cope with HIV-related symptoms [49]. There is also evidence that HIV service providers do not routinely address tobacco smoking. Crothers et al. [50] found that HIV providers may fail to recognize smoking in their patients at a higher rate than non-HIV medical providers. Further, less than half of surveyed New York State HIV/AIDS service providers always assessed tobacco smoking status or history among their patients [51]. Tobacco smoking is also not routinely addressed in mental health or harm reduction services. Indeed, in mental health or substance use treatment settings, smoking is sometimes encouraged as a harm reduction strategy, or an acceptable alternative to using illicit substances [52–54]. Based on a review of available literature, Prochaska [53] argued that this strategy runs contrary to the goals of these programs, in that tobacco smoking is associated with worse substance use treatment outcomes and increased depressive symptoms.

Many of the clients in this analysis (30 %) receive mental health and/or harm reduction services funded under Ryan White Part A. The NYC DOHMH has developed several initiatives to prioritize the issue of smoking among PLWH, including: (1) designating tobacco smoking reduction counseling as an eligible Part A service type (providers were previously not reimbursed for providing this service); (2) improving data collection to better track the prevalence, frequency, and type of tobacco use, in addition to smoking and tobacco use counseling services and referrals; (3) requiring at least one smoking and tobacco use counseling session every 6 months for harm reduction and mental health clients who report recent tobacco use on their latest substance use assessment form; and (4) offering smoking and tobacco use trainings to providers through the NYC DOHMH Bureau of Chronic Disease Prevention and Tobacco Control.

The following limitations should be taken into consideration when interpreting these findings. First, our data source on tobacco use is limited to the population of HIV-infected individuals who were receiving Ryan White Part A services in the NY EMA. Further, among these individuals, only those with at least one viral load and/or CD4 cell count test in the Registry within the 3 months prior to completing a substance use assessment were included in the analysis. Therefore, the findings should not be generalized to all PLWH in the NY EMA. Second, tobacco smoking and drug use were self-reported, and it is possible that social desirability bias influenced client responses to the substance use assessment. For example, clients may have feared that reporting substance use would jeopardize their ability to receive services or would lead to other discriminatory treatment by providers. Third, the data were cross-sectional; therefore, associations between recent cigarette smoking and HIV health outcomes should not be interpreted as evidence of causality. Fourth, there was a high prevalence of both outcome variables in the analysis population, in that 19 % (n = 2827) had a low CD4 cell count, and 36 % (n = 5346) had an unsuppressed viral load. These prevalence rates could have slightly inflated the resulting odds ratios. Finally, our analysis could not account for certain variables that could have influenced the relationship between recent tobacco smoking and biomarkers of HIV disease progression, including lifetime smoking status, smoking frequency, and ART adherence. While we were able to measure current ART prescription status, we could not assess date of ART initiation or whether a client was adherent to ART at the time of the substance use assessment. Due to the limitations of the form used to collect substance use information for this analysis, alcohol use was also not included as a covariate in this analysis, although the sensitivity analyses demonstrated that there was an independent relationship between recent tobacco smoking and unsuppressed viral load even among PLWH who reported no alcohol use in the last 3 months. Future studies should examine the possible influence of ART adherence and/or heavy alcohol consumption on the relationship between tobacco smoking and biomarkers of HIV disease progression in this population. This study, however, was strengthened by the use of the Registry, which is considered the most accurate and comprehensive source of HIV-related laboratory data on individuals diagnosed with HIV and/or receiving HIV care in New York City.

These results demonstrate a significant, independent association between recent tobacco smoking and biomarkers of HIV disease progression, including unsuppressed viral load and low CD4 cell count, in an HIV-service population. More research is needed to examine potential mechanisms that might explain the relationship between tobacco smoking and HIV health outcomes. This information is critical for the development of policy and practice for HIV/AIDS care, including the prioritization and planning of effective tobacco use screening tools and smoking cessation interventions.
